# Structure and Interactions of the TPR Domain of Sgt2 with Yeast Chaperones and Ybr137wp

**DOI:** 10.3389/fmolb.2017.00068

**Published:** 2017-10-11

**Authors:** Ewelina M. Krysztofinska, Nicola J. Evans, Arjun Thapaliya, James W. Murray, Rhodri M. L. Morgan, Santiago Martinez-Lumbreras, Rivka L. Isaacson

**Affiliations:** ^1^Department of Chemistry, King's College London, London, United Kingdom; ^2^Department of Life Sciences, Imperial College London, South Kensington, United Kingdom

**Keywords:** Sgt2, TPR, carboxylate clamp, NMR, CSP, x-ray crystallography, ITC

## Abstract

Small glutamine-rich tetratricopeptide repeat-containing protein 2 (Sgt2) is a multi-module co-chaperone involved in several protein quality control pathways. The TPR domain of Sgt2 and several other proteins, including SGTA, Hop, and CHIP, is a highly conserved motif known to form transient complexes with molecular chaperones such as Hsp70 and Hsp90. In this work, we present the first high resolution crystal structures of Sgt2_TPR alone and in complex with a C-terminal peptide PTVEEVD from heat shock protein, Ssa1. Using nuclear magnetic resonance spectroscopy and isothermal titration calorimetry, we demonstrate that Sgt2_TPR interacts with peptides corresponding to the C-termini of Ssa1, Hsc82, and Ybr137wp with similar binding modes and affinities.

## Introduction

Transient interactions between proteins confer functional versatility upon a range of cellular processes, including protein modification, transport, folding, and cell signaling pathways (Perkins et al., 2010). The co-chaperone, Small glutamine-rich tetratricopeptide repeat (TPR) protein alpha (SGTA), is involved in the decision to target various misfolded and mislocalized proteins into their appropriate pathways, upstream of either insertion to the endoplasmic reticulum (ER) or degradation (Hessa et al., 2011; Leznicki and High, 2012; Wunderley et al., 2014; Casson et al., 2016; Shao et al., 2017). SGTA interacts with many proteins and forms transient complexes with chaperones, membrane targeting proteins, and members of the ubiquitin/proteasome system (UPS; Rodrigo-Brenni et al., 2014; Leznicki et al., 2015; Krysztofinska et al., 2016; Thapaliya et al., 2016).

The yeast ortholog of SGTA, Sgt2, is best understood in the context of post-translational insertion of tail-anchored (TA) proteins into the ER membrane. The majority of membrane proteins undergo targeting to the endoplasmic reticulum in a co-translational process mediated by the signal recognition particle (SRP) as the nascent peptide chain emerges from the ribosomal tunnel. However, TAs are a special case of membrane proteins with obscured targeting signals at the extreme C-terminus. Therefore, their membrane delivery occurs post-translationally via the Guided Entry of Tail-anchored proteins pathway (GET; Schuldiner et al., 2008; Rabu et al., 2009; Borgese and Fasana, 2011; Hegde and Keenan, 2011). Sgt2 captures TA substrates after they are released from the ribosome and passes them on to the Get3 ATPase, the central targeting complex, in a process mediated by the Get4/Get5 heterodimeric scaffolding complex (Chartron et al., 2010; Wang et al., 2010; Simon et al., 2013; Mateja et al., 2015). This is followed by subsequent TA-protein release at the ER membrane, assisted by the Get1/Get2 heterodimeric membrane receptor complex (Wang et al., 2010; Mariappan et al., 2011; Vilardi et al., 2014). A new pathway in yeast has recently been discovered which is suggested to be the back-up mechanism in the event of GET system failure (Aviram et al., 2016). This involves three proteins, named Snd1, Snd2, and Snd3 (for SRP-independent targeting), which have possible roles in targeting substrates to the ER translocation machinery Sec61 (Aviram et al., 2016).

Importantly for its role, Sgt2 associates with several heat-shock proteins such as Hsp104, Hsc82 (yeast ortholog of Hsp90), and Ssa1/Ssa2 (yeast orthologs of Hsp70), which can bind directly to its central TPR domain (Liou and Wang, 2005). The Hsp70 and Hsp90 chaperones are important parts of the cellular machinery for protein folding, maturation and structural stability (Richter and Buchner, 2001; Pratt and Toft, 2003). They often associate with co-chaperones containing multiple copies of TPR domains (Frydman and Hohfeld, 1997; Pratt, 1997) which help them to facilitate correct folding of client proteins (Wang et al., 2010; Morgan et al., 2015). It has also been proposed that SGTA regulates the ATPase activity and folding rates of Hsp70 (Angeletti et al., 2002).

Recently, Sgt2 was reported to interact with Ybr137wp, a protein of uncharacterized function that is specific to yeast (Yeh et al., 2014). The Sgt2 TPR domain binds to the C-terminal end of Ybr137. Ybr137wp is thought to be a decamer both in its crystal form and in solution (Yeh et al., 2014). The function of Ybr137 is linked to the GET pathway where it is able to rescue the TA protein delivery-defect caused by a GET system that is impaired under starvation conditions (Yeh et al., 2014). However, the exact role for Ybr137wp in the TA targeting mechanism is not understood.

Sgt2 contains an N-terminal dimerization domain (Liou and Wang, 2005; Simon et al., 2013; Tung et al., 2013), followed by the conserved, central TPR domain and a glutamine rich region toward the C-terminus. The N-terminal domain of Sgt2 can directly bind the ubiquitin-like (UBL) domain of Get5 and facilitate the handover of TA substrates downstream onto GET pathway components for membrane delivery (Chartron et al., 2012; Simon et al., 2013; Darby et al., 2014). The C-terminal domain is predicted to be flexible based on SAXS experiments and, is structurally uncharacterized (Chartron et al., 2012). These domains of both Sgt2 and SGTA bind hydrophobic substrates including the TMDs of TA-proteins (Dutta and Tan, 2008; Wang F. et al., 2011; Leznicki et al., 2013). TPR domains typically consist of three or more tandem repeats of a loosely conserved 34 residue motif (Lamb et al., 1995; Smith, 2004). Each tandem motif is formed of two anti-parallel α-helices. TPR domains are well-known for mediating protein-protein interactions (Das et al., 1998). The structure of the human SGTA TPR domain was determined previously by X-ray crystallography (Dutta and Tan, 2008) and, like the yeast ortholog, has been reported to interact directly with Hsp70/Hsp90 chaperones, the proteasomal subunit Rpn13 and a variety of disease-related proteins (Buchanan et al., 2007; Dutta and Tan, 2008; Roberts et al., 2015; Thapaliya et al., 2016). Moreover, the TPR domain structure (including some additional linker residues at the C-terminal) of the Sgt2 homolog from *Aspergillus fumigatus* was solved by crystallography (Chartron et al., 2011).

Several TPR domains of various proteins are known to interact with Hsp70/Hsp90 peptides. The high-resolution structures of such complexes were reported for HOP TPR1 with an Hsp70-derived peptide, HOP TPR2A with an Hsp90-derived peptide (Scheufler et al., 2000) and CHIP TPR with an Hsp70 C-terminal peptide (Zhang et al., 2005; Wang Q. et al., 2011). The common mode of interaction involves the formation of a carboxylate clamp where both the side-chain and main-chain terminal carboxylate groups of the C-terminal aspartic acids of these peptides form salt bridges with conserved arginine residues within the groove of the cochaperone TPR domains. Currently there is no structure of the Sgt2 TPR domain from *Saccharomyces cerevisiae*.

In this study, we report the first high resolution X-ray structure of the Sgt2_TPR at 1.55 Å and also in complex with the PTVEEVD peptide corresponding to the C-terminus of Ssa1, at 2.0 Å. In addition, we have characterized the interaction between Sgt2_TPR and C-terminal protein fragments of Ssa1/Ssa2 and Hsp82 (Hsp70 and Hsp90 in mammals respectively) and Ybr137wp using isothermal titration calorimetry (ITC) and Nuclear magnetic resonance (NMR).

## Results

### Overall Sgt2_TPR structure

**Figure 1:**
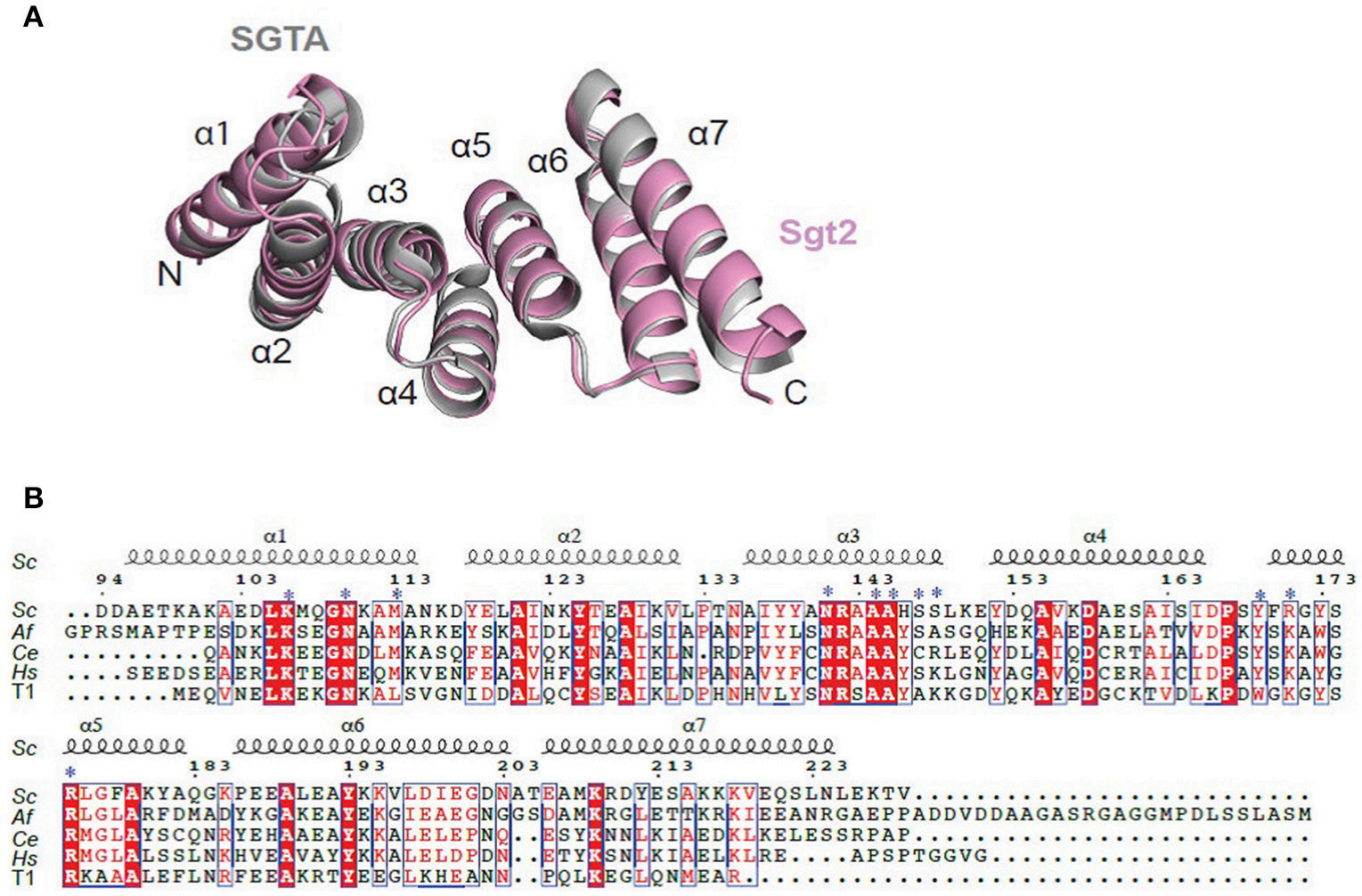
Crystal structure of Sgt2_TPR. **(A)** Superimposition of TPR domains from Sgt2 (magenta, PDB: 5LYN) with human SGTA (gray, PDB: 2VYI) Structures were superposed using secondary-structure matching in ccp4mg (McNicholas et al., 2011). The structures align with RMSD of 1.13 Å over 135 C_α_. **(B)** Structure-based sequence alignment of SGT TPR domains from *S.cerevisiae (Sc), A.fumigatus (Af), C. elegans (Ce), Homo sapiens (Hs)*, and human HOP TPR1 (T1). The residues involved in interaction and forming the two-carboxylate clamp are indicated with asterisks; boxes show conserved residues while red highlights sequence identity, structural motifs are labeled on the top. The residue numbering is from *S.cerevisiae*. Figure generated using ESPript 3.0 server.

The structure of Sgt2_TPR was determined by molecular replacement and refined to 1.55 Å resolution (Figure 1A, PDB: 5LYP; data collection and refinement parameters in Table 1). The coordinates of SGTA_TPR (PDB: 2VYI) were used as a search model since there is high structural homology with Sgt2_TPR (57% sequence identity; Figure 1B).

**Table 1:** Data collection and refinement statistics of Sgt2_TPR.

Resolution range	32.31–1.55 (1.605–1.55)	CC (work)	0.939 (0.928)
Space group	P 21 21 21	CC (free)	0.921 (0.863)
Unit cell	a: 36.86Å, b: 50.76Å c: 67.12 Å α: 90°, β: 90°, γ: 90°	Number of non-hydrogen atoms	1,157
Total reflections	232,024 (23272)	Macromolecules	1,062
Unique reflections	18,908 (1864)	Ligands	5
Multiplicity	12.3 (12.5)	Protein residues	137
Completeness (%)	1.00 (1.00)	RMS (bonds)	0.005
Mean I/sigma(I)	12.89 (8.72)	RMS (angles)	0.62
Wilson B-factor	8.98 Å^2^	Ramachandran favored (%)	100
R-merge	0.2183 (1.061)	Ramachandran allowed (%)	0
R-meas	0.2277 (1.104)	Ramachandran outliers (%)	0
CC1/2	0.995 (0.975)	Rotamer outliers (%)	0.95
CC^*^	0.999 (0.994)	Clashscore	0.95
Reflections used in refinement	18,908 (1,864)	Average B-factor	10.96 Å^2^
Reflections used for R-free	937 (89)	Macromolecules	10.50
R-work	0.1936 (0.1711)	Ligands	19.64
R-free	0.2186 (0.2231)	Solvent	15.95

*Statistics for the highest-resolution shell are shown in parentheses*.

CC^*^ is a derived quantity that links data and model and estimates the correlation of an observed data set with the underlying true signal.

All residues were built into the electron density map except for the C-terminal Val229 and the solvent-exposed sidechains of Glu93 and Asp94. The final model also contains 90 water molecules and a single BO_4_ ion from the crystallization condition. The TPR domain of Sgt2 consists of three TPR repeats, comprising six almost identical α-helices and a C-terminal “capping” helix (α1 = A96-N115; α2 = Y118-V131; α3 = A136-L149; α4 = Y152-I165; α5 = F170-183Q; α6 = P186-E200; α7 = E206-L225) connected by short loops and arranged in a antiparallel fold homologous to that of SGTA_TPR. A structural overlay with the equivalent human domain is shown in Figure 1A (RMSD of 1.13 Å over 135 C_α_).

### Complex structure of Sgt2_TPR with the C-terminal peptide of Ssa1

**Figure 2:**
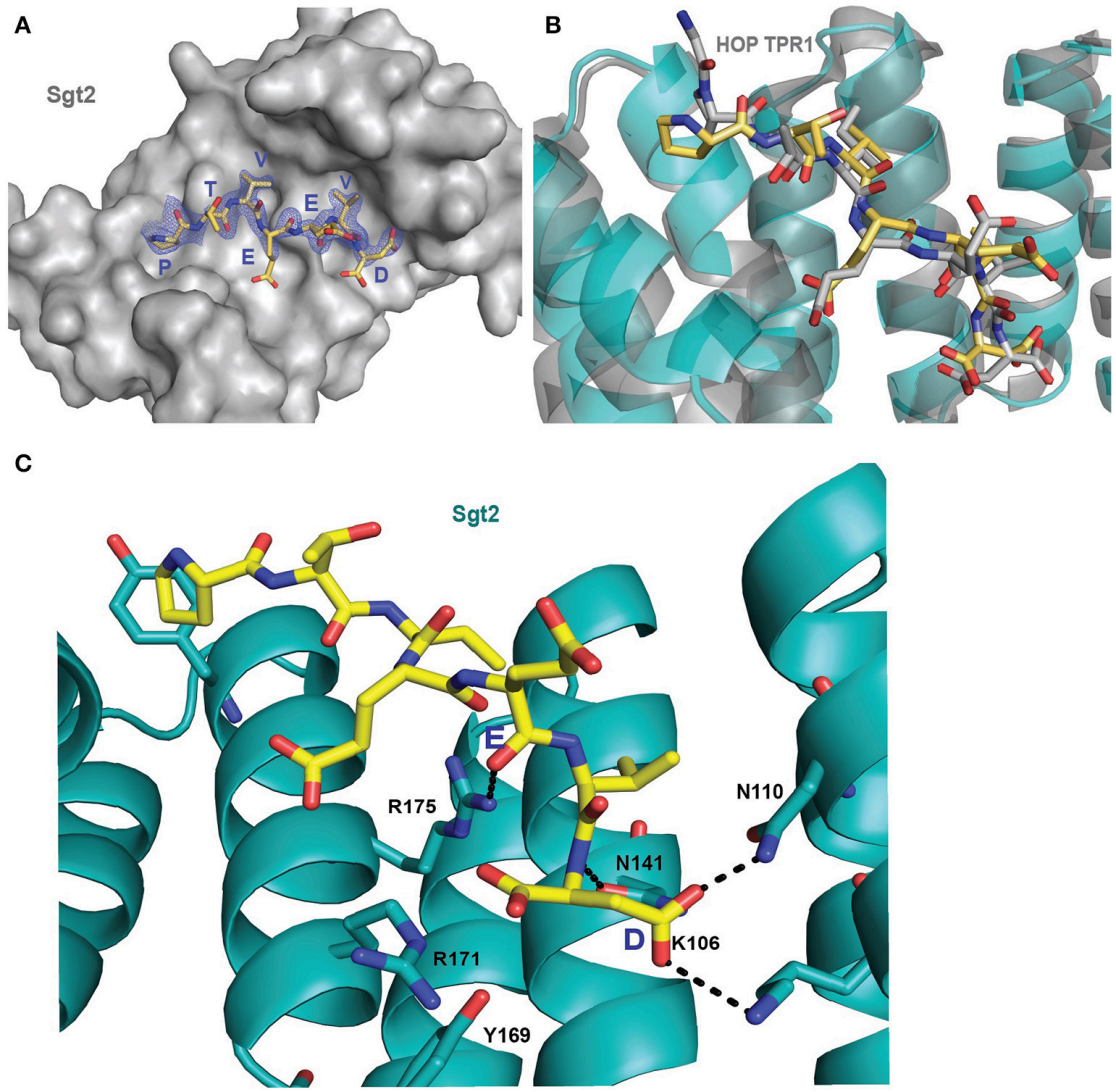
Crystal structure of Sgt2_TPR/PTVEEVD complex. **(A)** The surface representation of Sgt2 hydrophobic groove with bound Ssa1 derived PTVEEVD peptide (PDB: 5LYP). The 2Fo-Fc map for the peptide was contoured at 1.0 σ. **(B)** Superimposition of Sgt2_TPR/PTVEEVD complex (peptide in yellow) onto Hsc70 peptide-bound HOP TPR1A (peptide in gray, PDB: 1ELW) highlighting similarities in peptide conformation at the binding interface. The peptides align with RMSD 0.52 Å. **(C)** Network of interactions formed at the complex interface (chain A and D). Residues shown as sticks are involved in the formation of hydrogen bonds or electrostatic interactions (shown as black dashed lines). Residues K106, N110, N141, R171, R175, and Y169 are involved in the formation of two-carboxylate clamp and M113 is involved in hydrophobic interactions.

Initially it was not possible to form a crystal complex of Sgt2_TPR (93–229) with the C-terminal of Ssa1 due to the flexible N-terminal and C-terminal ends of symmetry-related molecules in the crystal occluding the binding interface. However, producing a shorter Sgt2_TPR (96–225) construct by removing three residues (EDD) from the N-terminus and four (EKTV) from the C-terminus resulted in successful crystallization of the Sgt2_TPR/PTVEEVD complex (Figures 2A–C, PDB: 5LYN, statistics in Table 2).

**Table 2:** Data collection and refinement statistics of Sgt2_TPR complex.

Resolution range	33.59–2.0 (2.071–2.0)	CC (work)	0.845 (0.345)
Space group	P 1 21 1	CC (free)	0.725 (0.476)
Unit cell	a: 45.49Å, b: 61.09Å c: 55.25Å, α: 90°, β: 108.81°, γ: 90°	Number of non-hydrogen atoms	2,340
Total reflections	117,547 (10,486)	Macromolecules	2,183
Unique reflections	19189 (1,904)	Ligands	9
Multiplicity	6.1 (5.6)	Protein residues	280
Completeness (%)	0.99 (0.97)	RMS (bonds)	0.006
Mean I/sigma(I)	6.49 (2.84)	RMS (angles)	0.68
Wilson B-factor	25.49 Å^2^	Ramachandran favored (%)	99.63
R-merge	0.354 (0.8515)	Ramachandran allowed (%)	0.37
R-meas	0.3851 (0.9392)	Ramachandran outliers (%)	0
CC1/2	0.92 (0.531)	Rotamer outliers (%)	0
CC^*^	0.979 (0.833)	Clashscore	2.77
Reflections used in refinement	19,279 (1903)	Average B-factor	30.91 Å^2^
Reflections used for R-free	928 (83)	Macromolecules	30.60
R-work	0.1576 (0.1903)	Ligands	50.77
R-free	0.2025 (0.2587)	Solvent	34.38

*Statistics for the highest-resolution shell are shown in parentheses*.

CC^*^ is a derived quantity that links data and model and estimates the correlation of an observed data set with the underlying true signal.

Two copies of the TPR domain were present in the asymmetric unit due to non-crystallographic symmetry (Figure S1A). All Sgt2_TPR residues could be built into electron density maps in chain A and chain B. The two peptides (chains C and D) were partially occupied and were built with care. They were modeled into electron density (Figure 2A) and then verified by producing a simulated annealing omit map (Figure S2). The overall structures of the two chains, A and B, are very similar in their backbones (RMSD of 0.77 Å over 135 C_α_), with deviations observed for the R171 sidechains possibly due to their flexibility and significant differences in the modeled C and D peptides (Figures S1A,B). The electron density was ambiguous for peptide C at chain B, especially for Pro1 with some electron density appearing in the Fo-Fc map which could be suggesting the presence of another atom. However, neither a zinc ion nor water molecule could be fitted. Nevertheless, all PTVEEVD (1–7) residues were successfully modeled. The occupancy for the peptide chains (C and D) was refined to an Rfactor of 0.158 and an Rfree of 0.202, and both converged to occupancy 0.93. The final model also contained 148 water molecules, nine Zinc ions and a single BO_4_ ion, which was present in the M9 medium we used for protein expression. Zinc ions were added at peaks of the phased anomalous difference map (DANO).

The interaction between the Sgt2_TPR and the PTVEEVD peptide from Ssa1 is mostly driven by the formation of a two-carboxylate clamp. Most of the electrostatic interactions between the TPR domain and the peptide occur in the C-terminal EEVD region and anchor the peptide in place. Peptide chains C and D, whilst overlapping, show slight conformational differences with an RMSD of 1.24 (Figure 2B). PDBePISA highlights this difference showing a binding surface area of 474.7 Å^2^ between chain B and C, and 514.3 Å^2^ between chain A and D, and a difference in solvation energy of binding of −0.8 ΔiG kcal/mol and −3.7 ΔiG kcal/mol respectively. In the interface between chain A and D direct backbone contacts involve hydrogen bond formation between the carboxamide sidechains of Asn141 and Asn110 in Sgt2_TPR, and the sidechain and backbone of the terminal Asp7 of the Ssa1 peptide. Moreover, the sidechain amine of Lys 106 binds to the same carbonyl sidechain of Asp7. The guanidinium group of Arg171 forms a salt-bridge with the carbonyl main chain of Asp7 and forms an additional internal contact with Tyr169. The Arg175 sidechain interacts with the carbonyl main chain of Glu4 and Glu5 of the peptide (Figure 2C). In addition, the N-terminal of the peptide is involved in hydrophobic and van der Waals interactions. Phe178 and Tyr181 contribute to creating hydrophobic pockets and interact with the aliphatic part of the Pro1, Thr2, and Val3 residues. Met113 makes a hydrophobic contact with Val6 of the peptide (Figure S3). The “two-carboxylate clamp” binding mode is characteristic for TPR domains interacting with the conserved C-terminal IEEVD and MEEVD motifs of Hsp70 and Hsp90 chaperones, respectively (Scheufler et al., 2000; Zhang et al., 2005). Comparing the Sgt2_TPR/PTVEEVD binding interface with a previously published complex of HOP TPR1/GPTIEEVD (Hsp70-derivative; Scheufler et al., 2000) shows that the PTVEEVD peptide occupies the same position at the Sgt2 TPR groove as GPTIEEVD. The peptides overlap apart from a difference in the conformation of the main chain and sidechain of the terminal Asp7 of GPTIEEVD (Figure 2B). The alternative Asp7 conformation however overlaps with the Asp7 conformation in our chain C peptide in the complex structure. Comparing the two copies of the TPR-bound peptide indicates that, in the interface between chains B and C, the main difference is in the backbone of the N-terminal of peptide C. This is manifested through small variations in the orientation of Pro1, Thr2 and Val3 sidechains, changing hydrophobic interactions (particularly between Val3 and Phe178 of the TPR), and new electrostatic interactions between Thr2 and Asp211. These differences are not driven by the binding interface of Sgt2_TPR, as the sidechains of A and B largely adopt the same conformation, except for Arg171 due to its inherently flexible sidechain (Najmanovich et al., 2000). Notably, the Arg171 backbone NH was missing in the HSQC spectrum, which is a common feature of flexible sidechains in intermediate exchange on NMR timescales. The change in orientation of chain B Arg171, creates a salt bridge with the sidechain of Glu4. In addition, slight conformational changes in chain C Asp7 facilitate formation of hydrogen bond between the hydroxyl group of Tyr169 and the Asp7 peptide sidechain and the interaction between the sidechains of Asn141 and Asn110 of Sgt2_TPR and the main chain of Asp7 (Figure S1B).

### Interactions of the TPR domain of Sgt2 with yeast chaperones and Ybr137wp

**Figure 3:**
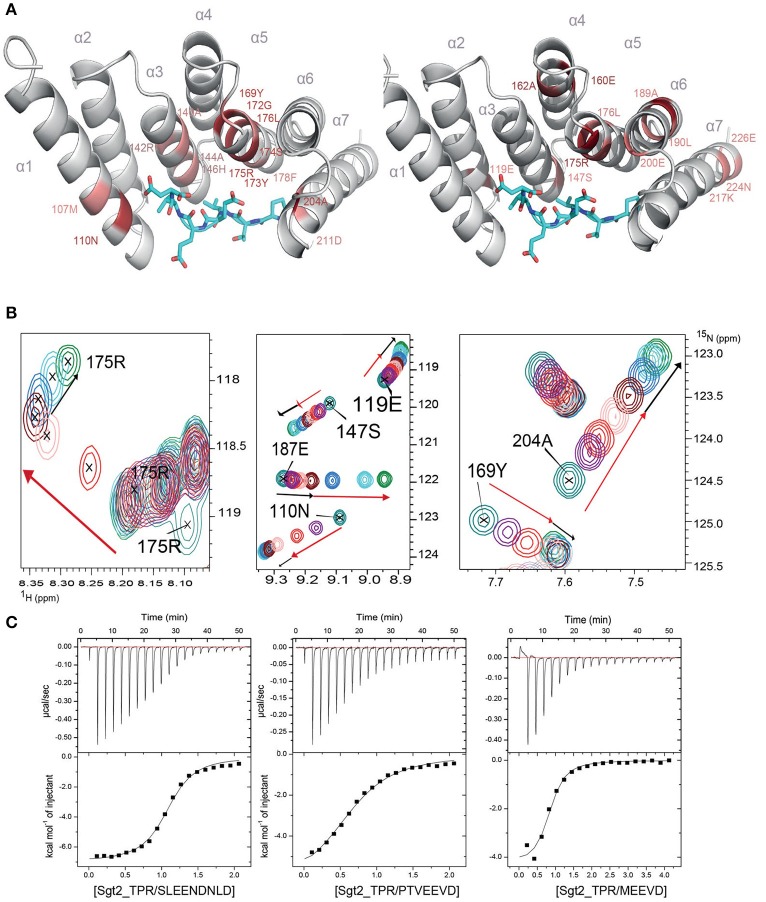
Sgt2_TPR binds yeast chaperones and Ybr137wp. **(A)** Sgt2_TPR domain colored according to reciprocal chemical shift perturbation (CSP) upon additions of unlabeled PTVEEVD peptide. The model on the left shows the titration points between 0 and 1:3 protein/peptide molar ratio and on the right between 1:3 and 1:6 molar ratio. The most perturbed residues were selected at >0.4 ppm (red) and >0.3 ppm (light red) for the model on the left and >0.2 (red) and >0.15 (light red) for the model on the right. **(B)** A small regions of the ^1^H-^15^N HSQC spectrum of ^15^N-labeled Sgt2_TPR titrated with unlabeled PTVEEVD at ratios: 1:0 (teal), 1:0.5 (purple), 1:1 (red), 1:2 (light pink), 1:3 (maroon), 1:4 (blue), 1:5 (cyan), and 1:6 (green). Black and red arrows indicate the first and second events in CSPs upon titration respectively. **(C)** ITC data showing binding of Sgt2_TPR to SLEEDLNLD, PTVEEVD, and MEEVD. Binding parameters, determined by ITC were K_d_ = 1.53 ± 0.05 μM, ΔH = −7.07 ± 0.99 kcal/mol; ΔS = 3.19 kcal/mol·deg for SLEEDLNLD; K_d_ = 9.04 ± 0.05 μM, ΔH = −6.72 ± 0.07 kcal/mol, ΔS = 140 cal/mol·deg for PTVEEVD and K_d_ = 2.95 ± 0.31 μM, ΔH = −4.51 ± 0.03 kcal/mol; ΔS = 10.9 cal/mol·deg, for MEEVD.

To understand the molecular details of the interactions between Sgt2_TPR and the C-terminal fragments of Ssa1 (PTVEEVD), Hsp82 (MEEVD), and Ybr137wp (SLEEDLNLD) we also performed NMR and ITC experiments. We acquired a complete set of NMR triple resonance experiments using the longer construct of Sgt2_TPR (93–229) to facilitate the full backbone assignment (BMRB Accession Number: 27044). All residues were assigned except for Arg171. Reciprocal chemical shift perturbation (CSP) experiments were carried out by titrating the unlabeled peptides PTVEEVD, MEEVD and SLEEDLNLD into ^15^N-labeled Sgt2_TPR (93–229) up to a six-fold molar excess of PTVEEVD and MEEVD and a five-fold excess of SLEEDLNLD. The NMR backbone assignment of Sgt2_TPR allowed us to identify the residues involved in the interactions in all three titrations (Figure S4, S5A, S6A). The CSP analysis exhibited a similar pattern for all three peptide titrations (Figure S7) and showed binding in a fast exchange regimen, with a number of peaks shifting non-linearly suggesting the formation of an intermediate during the titration. We analyzed the Sgt2_TPR CSPs (Figures 3A–C) by applying a titration cut-off at 3 molar equivalents of the peptide for all peaks and then dividing them into two groups. The first group consisted of peak shifts between 0 and 3 molar equivalents and was named “first event,” and the second group, called “second event,” comprised peak shifts that occurred between 3 and 6 (or 5 in the case of SLEEDLNLD) molar equivalents (marked as red and black arrows respectively in Figure 3B, Figure S5B, S6B). The CSP of Sgt2_TPR/PTVEEVD binding for the “first event,” shown on our x-ray structure is shown in Figure 3A on the left, and the “second event” on the right. The most perturbed resides for the “first event” correspond to the residues at the binding interface in the x-ray complex structure and/or the neighboring residues.

We also characterized the binding between Sgt2_TPR (93–229) and PTVEEVD, MEEVD, and SLEEDLNLD peptides by ITC (Figure 3C). The ITC results indicate a similar binding affinity for all three complexes with dissociation constants (K_d_) of 9.04 ± 0.05 μM for Sgt2_TPR/PTVEEVD, 2.95 ± 0.30 μM for Sgt2_TPR/MEEVD and 1.53 ± 0.05 μM for Sgt2_TPR/SLEEDLNLD. The favorable enthalpy and entropy values obtained from ITC suggest that all complex formations were driven by the establishment of both hydrogen bonds and hydrophobic interactions (Figure 3C).

## Discussion

In this study we provide high-resolution X-ray structures of the free Sgt2_TPR domain and its complex with the last seven amino acids of Ssa1 (Hsp70). We also assign the backbone of Sgt2_TPR using NMR spectroscopy and characterize the interaction between Sgt2_TPR and the extreme C-terminal fragments of Hsc82 (Hsp90) and Ybr137wp. Our structural data clearly show that Ssa1 binds to the TPR domain of Sgt2 via a carboxylate clamp mechanism and we can predict a similar mode of binding for Hsc82 and Ybr137wp from our consistent ITC and NMR data in all three systems. Analysis of the three protein-peptide complexes using the PDBePISA interactive tool indicates similarities. In all three complexes Glu4 and Glu5 are predicted to be involved in formation of hydrogen bonds and the terminal, Asp7, can form a salt bridge with Arg171, Arg175, and Lys106. In addition, in the case of the SLEEDLNLD peptide there is potential for Glu3 to also be involved in a hydrogen bond formation with Ser148. There is a binding surface area of 514.3 Å^2^ between chains A and D, and a difference in solvation energy of binding of −3.7 ΔiG kcal/mol with PTVEEVD. In modeled examples of MEEVD and SLEEDLNLD there are binding surface areas of 420.8 and 579.5 Å^2^, and differences in solvation energy of binding of −2.7 ΔiG kcal/mol and −2.2 ΔiG kcal/mol, respectively. There are many examples in the literature of carboxylate clamp mechanisms, most of them connecting the C-termini of heat shock proteins with different co-chaperones (Carrigan et al., 2004; Prasad et al., 2010; Panigrahi et al., 2014), but there are also non-chaperone examples which include the recognition of the proteasomal protein Rpn13 by SGTA (Thapaliya et al., 2016) and the interaction of Sgt2 with Ybr137wp presented here.

All carboxylate clamp interactions studied so far, including our recent and past investigations, describe dissociation constants in the low micromolar range by ITC or SPR (Scheufler et al., 2000; Brinker et al., 2002; Worrall et al., 2008; Thapaliya et al., 2016). In addition our NMR data suggest the presence of an intriguing dual-event binding mode during titrations and a widespread perturbation along the whole TPR domain. A detailed analysis of the titration revealed that in the first event only residues in the binding interface appear perturbed, while in the second event the perturbation is not localized to a specific interface. A similar scenario had previously been observed in the Rpn13 interaction with SGTA TPR, where signals all over the TPR were affected upon titration (Thapaliya et al., 2016). This behavior appears conserved for carboxylate clamp recognition whether synthetic peptides or recombinant proteins were used for the titration experiments. It likely relates to the fact that the TPR domain helices suffer a subtle contraction to enclose the peptide in the TPR groove. The crystallographic structures we have obtained clearly show a slightly more compact conformation of the TPR where helices 1 and 7 are closer to each other in the complex structure than in the unbound TPR (See Figure S8 for a structural alignment). This observation was previously reported for a longer TPR motif (Zeytuni et al., 2011) and proposed for the TPR domains of co-chaperones (Panigrahi et al., 2014), where it was suggested that changes in the curvature of the cradle structure by concerted movement of the helices may be necessary for ligand binding.

The orientation of Hsp peptides in TPR structures varies between proteins. The conserved clamp mode of interactions is consistent, but there are some differences observed for the N-terminal parts of the peptides, which is not surprising given that the carboxylate clamp is the fixed point of attachment. The PTVEEVD peptide adopts an extended conformation within the Sgt2_TPR groove similar to that observed in the structure of the HOP TPR1/GPTIEEVD complex (Scheufler et al., 2000). In contrast, structures of GPTIEEVD/Chip TPR [PDB: 3Q49 (Wang L. et al., 2011) and PDB: 4KBQ Zhang et al., 2005] show the peptide in a curled conformation lining the groove. The structures also vary in orientations of the peptide Pro1. In comparison, we also observe differences between the Pro1, Thr2, and Val3 sidechains in the two chains of our Sgt2_TPR/PTVEEVD complex structure allowing for some flexibility in the association between TPR and Hsp peptides at the same interface. Sgt2_TPR serves as a binding interface for transient interactions with a variety of chaperones and other proteins. However, the extended conformation of the peptides and their position within the TPR groove allows for a widespread contact surface with TPR domains thus supporting the specific recognition of short amino acid stretches with sufficient affinity to bind (Figure S6). The preceding residues to EEVD are also important for the binding affinity and it has been reported that trimming the peptide sequence to EEVD only, reduced the affinity by at least 10 times (Scheufler et al., 2000). Furthermore, Sgt2 is a homodimer and it can target a broad range of substrates by binding more than one protein simultaneously and bringing them into closer proximity promoting interactions.

Little is known about the role of Ybr137wp in the GET pathway except that it binds to Sgt2 at the same binding interface as heat shock chaperones and that it can influence TA membrane insertion by mediating interactions between Sgt2 and chaperones. Previous ITC binding experiments reported that one full-length Ybr137w decamer is capable of binding to five Sgt2_TPR dimers with a dissociation constant (K_d_) of 1.38 ± 0.09 μM (Yeh et al., 2014). This is almost identical to the ITC results we obtained for the association of Sgt2_TPR with the extreme C-terminal nine-residue Ybr137wp-derived peptide (K_d_ of 1.53 ± 0.05), suggesting that the SLEEDLNLD fragment is sufficient for the interaction. Moreover, it has been shown that removing ESLEEDLNLD from the C-terminal of Ybr137wp abolished the interaction, confirming that this flexible C-terminal region is also necessary for the interaction (Yeh et al., 2014).

Further work is required to define the distinct role of Ybr137wp in ER delivery of tail-anchored membrane proteins and examine whether there is any link between this protein and the recently discovered SND targeting pathway in yeast. This alternative to the GET and SRP mechanisms, is proposed to act as a back-up targeting system (Aviram et al., 2016). It involves three proteins, localized at the ER or in the cytoplasm, Snd1 (encoded by YDR186C), Snd2 (encoded by ENV10, also known as YLR065C) and Snd3 (encoded by PHO88, also known as YBR106W), working together in a joint targeting pathway (Aviram et al., 2016). The function of Ybr137wp is also linked with altering the defect in TA protein delivery and cell viability derived from impairment of the GET system under starvation conditions.

Future investigations will improve our understanding of Ybr137wp function which will shed light on the importance of the carboxylate clamp interaction with Sgt2 that we delineate here.

## Methods

### Plasmid preparation

Gene fragments encoding the Sgt2_TPR (residues 93–229 and 96–225 for the shorter construct) from *S. cerevisiae* were PCR amplified from synthetic cDNA (Life Technologies) and cloned into the *Bam*HI/*Xho*I restriction sites of a home-modified pET28 vector which encodes an N-terminal thioredoxin A fusion protein followed by a hexahistidine tag and tobacco etch virus (TEV) protease cleavage site.

### Protein production

All plasmids carrying Sgt2_TPR were transformed into *E. coli* BL21 (DE3) strain. Typically, protein expression was induced by adding 0.3–0.5 mM isopropyl-β-D-thiogalactopyranoside (IPTG) to cultures at OD_600_ ≈ 0.8, followed by overnight incubation at 18°C. For ^15^-N-labeled proteins, growth was carried out in M9 media supplemented with labeled ammonium chloride (>98 % ^15^N, Sigma-Aldrich) and/or glucose (>99% U-^13^C, Sigma-Aldrich). Harvested cells were resuspended in lysis buffer [20 mM potassium phosphate, pH 8.0, 300 mM NaCl, 10 mM Imidazole, 250 μM tris(2-carboxyethyl)phosphine (TCEP)], supplemented with 1 mM phenylmethylsulfonyl fluoride (PMSF), and lysed by sonication or using a cell disruptor (Constant Systems Ltd). Cell debris and insoluble material were removed by centrifugation and overexpressed protein recovered from soluble fractions was purified using nickel affinity chromatography (HisTrap^TM^ HP 5 ml, GE Healthcare). Recombinant proteins were eluted with buffer containing 300 mM imidazole, then dialyzed against cleavage buffer (20 mM potassium phosphate, pH 8.0 and 300 mM NaCl) and digested with homemade TEV protease (≈100 μ g/ml) at 4°C overnight. After TEV cleavage, a second nickel affinity chromatography step was performed to remove fusion protein, histidine tags, undigested protein, and TEV protease; the desired protein was then recovered in the flow through and loaded into a HiLoad 16/60 Superdex 75 column (GE Healthcare), previously equilibrated in buffer containing 10 mM potassium phosphate pH 6.0, 100 mM NaCl and 250 μ M TCEP or 20 mM Tris-HCl pH 7.5. Proteins were concentrated using Vivaspin concentrators with 5K cut-off (Sartorius Stedin) and sample purity and homogeneity was checked by SDS-PAGE, mass spectrometry and NMR. The lyophilized peptides: PTVEEVD (corresponding to Ssa1 C-terminal; residues 634–640), MEEVD (corresponding to C-terminal of Hsp82; residues 705–709) and SLEEDLNLD (corresponding to C-terminal of Ybr137wp; residues 171–179) were purchased from Alpha BioScience (Birmingham, UK) and resuspended in water or an appropriate buffer before use. All peptides were purified and verified by HPLC and mass spectrometry.

### NMR titrations

Sgt2_TPR (residues 93–229) and peptides used for NMR were prepared in 10 mM potassium phosphate pH 6.0, 100 mM NaCl and 250 μ M TCEP buffer. Typically, ^1^H-^15^N HSQC experiments were recorded for each titration point at 25°C and CSP calculated for each amide signal using the following formula, where Δ δ _1H_ and Δ δ _15N_ are the chemical shift differences for the same amide in its free and bound spectra (δ _free_-δ_bound_) and for proton and nitrogen values respectively:

$$\begin{array}{l} {\Delta \delta }^{av}=\sqrt{\left({\left(\Delta \delta_{1H}\right)}^{2}+{\left(\Delta \delta_{15N}/5\right)}^{2}\right)\cdot 0.5} \end{array}$$

CSP results were mapped onto the structures using the PyMOL software.

### NMR experiments

Protein samples at concentrations between 500 and 3,000 μM were prepared in 10% D_2_O (Sigma Aldrich), 10 mM potassium phosphate pH 6.0, 100 mM NaCl and 250 μM TCEP buffer. All NMR experiments were acquired in 5 mm NMR tubes at 25°C on Bruker Avance spectrometers operating at 500 and 800 MHz equipped with cryoprobes, controlled by the TopSpin 3.1 software package. Backbone assignments were carried out using 3D experiments [HNCO, HNCA, HN(CA)CO, CBCA(CO)NH, and CBCANH] for Sgt2_TPR. All NMR spectra were processed with NMRPipe (Delaglio et al., 1995) and analyzed with CcpNMR Analysis (Vranken et al., 2005).

### ITC

ITC measurements were performed at 25°C using an ITC-200 MicroCal microcalorimeter (GE Healthcare) following standard procedures (Darby et al., 2014). Proteins were prepared in 10 mM potassium phosphate pH 6.0, 100 mM NaCl, 250 μM TCEP. In each titration, 20 injections of 2 μL of peptide solution at a concentration of 500 μM, were added to Sgt2_TPR (residues 93–229) at 50 μM in the reaction cell. Integrated heat data obtained for the titrations, corrected for heats of dilution, were fitted using a nonlinear least-squares minimization algorithm to a theoretical titration curve, using the MicroCal-Origin 7.0 software package. ΔH (reaction enthalpy change in Kcal/mol), K_b_ (equilibrium binding constant per mole), and n (molar ratio between the proteins in the complex) were the fitting parameters. The reaction entropy, ΔS, was calculated using the ΔG = −RT·lnK_b_ (*R* = 8.314 J/(mol·K), T 298 K) and ΔG = ΔH −T ΔS. Dissociation constants (*K*_d_) are shown in the figure legends for each interaction.

### Crystallization

Sgt2_TPR (residues 93–229) was concentrated to 35 mg/ml in 20 mM Tris-HCl pH 7.5 buffer and crystals were obtained after 4 days by the vapor-diffusion method at 293K using MRC plates in 0.1 M SPG, pH 6.0, 25% w/v PEG 1500 (PACT premier from Molecular Dimensions) at 20°C (drop volume = 400 nl). In the case of Sgt2_TPR (96–225)/PTVEEVD complex, protein/peptide complex was eluted from a HiLoad 16/60 Superdex 75 column and concentrated to 20 mg/ml in 20 mM Tris-HCl pH 7.5 followed by a further peptide addition (up to protein: peptide molar ratio of 1:3) prior to crystallization. The complex crystalized after 7 days by the vapor-diffusion method at 293 K in 0.2 M zinc Acetate, pH 7.2, 30% w/v PEG 3350. All crystals were harvested in reservoir solution with 20% glycerol before flash cooling in liquid nitrogen.

### Data collection and processing

A complete dataset was collected from a single crystal on Diamond Beamline I04 for the free Sgt2_TPR dataset and I03 for the Sgt2_TPR/PTVEEVD complex using a Pilatus 6M-F detector and a single wavelength 0.920 Å. Data were processed using Xia2 (Winter et al., 2013) with scaling and merging using Aimless (McNicholas et al., 2011).

### Structure solution and refinement

The crystal structure of Sgt2_TPR (93–229) was determined by molecular replacement using Phaser (McCoy et al., 2007) with the human SGTA_TPR crystal structure (PDB: 2VYI) used as a search model (57% sequence identity). This structure was then used as the search model to solve the Sgt2_TPR (96–225)/PTVEEVD complex. Both structures were refined using REFMAC5 (Murshudov et al., 2011) and PHENIX (Adams et al., 2010) with manual model building using Coot (Emsley and Cowtan, 2004). Free R-value of 4.9% was used as a cross-validation method for Sgt2_TPR and 4.8% for Sgt2_TPR (96–225)/PTVEEVD. Water molecules, Zn atoms and BO_4_ atoms were fitted manually using Coot. The free Sgt2_TPR structure was solved in space group P 21 21 21 with cell parameters: 36.86 Å (a) 50.76 Å (b) 67.12 Å (c) 90.00° (α) 90.00° (β) 90.00° (γ). The refined structure shows very good stereochemistry (statistics from the Molprobity (Chen et al., 2010, 2015) report are shown in Table 1). The complex structure was solved in space group P21 with cell dimensions: 45.49 Å (a) 61.09 Å (b) 55.25 Å (c) 90.00° (α)108.81° (β) 90.00° (γ) and statistics from the Molprobity (Chen et al., 2010, 2015) report are shown in Table 2).

## Author contributions

EK, NE, AT, and RI conceived the ideas and designed experiments. EK, NE, and AT performed experiments. EK, NE, AT, JM, RM, SM, and RI analyzed data. All authors contributed toward writing the manuscript.

### Conflict of interest statement

The authors declare that the research was conducted in the absence of any commercial or financial relationships that could be construed as a potential conflict of interest.
