# Supplementary material for: Structure and Interactions of the TPR Domain of Sgt2 with Yeast Chaperones and Ybr137wp
Source: Front Mol Biosci. 2017 Oct 11;4:68. doi: 10.3389/fmolb.2017.00068 (PMC5641545; doi:10.3389/fmolb.2017.00068)
Supplement: Supplementary file 1 [file DataSheet1.docx]

**Supplementary Material**


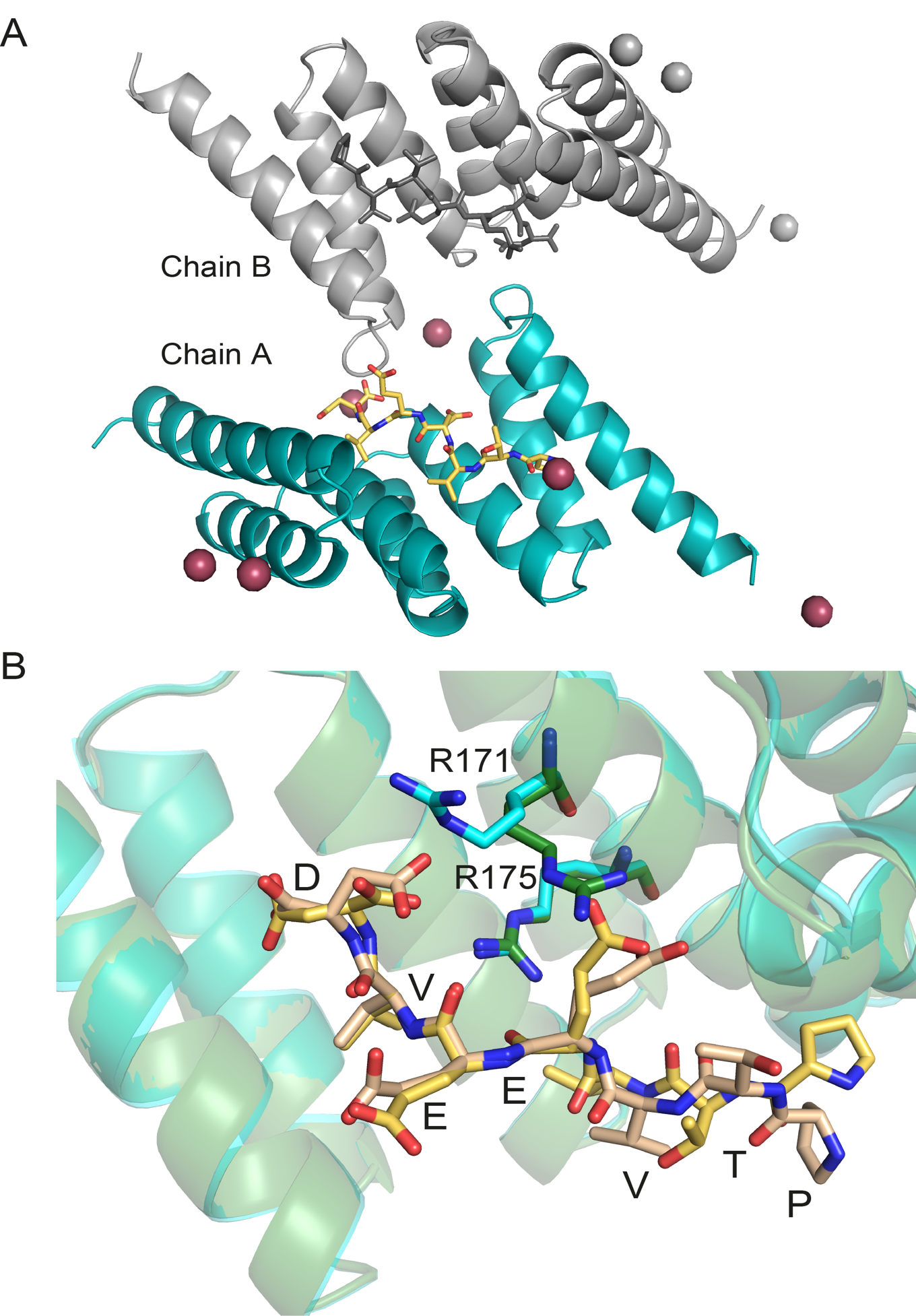


**Figure S1:** A) The asymmetric unit shown as cartoon representation of Sgt2_TPR chain A (teal) and chain B (grey) and bound peptides (yellow and grey respectively). Zinc ions showed as balls. B) Superimposition of Sgt2_TPR Chain A (teal) and Chain B (green) bound peptides (yellow and wheat respectively) highlighting main differences. Peptides were superposed using secondary-structure C_α_ matching in ccp4mg^56^. The peptides align with RMSD 1.24 Å over 7 C_α_.


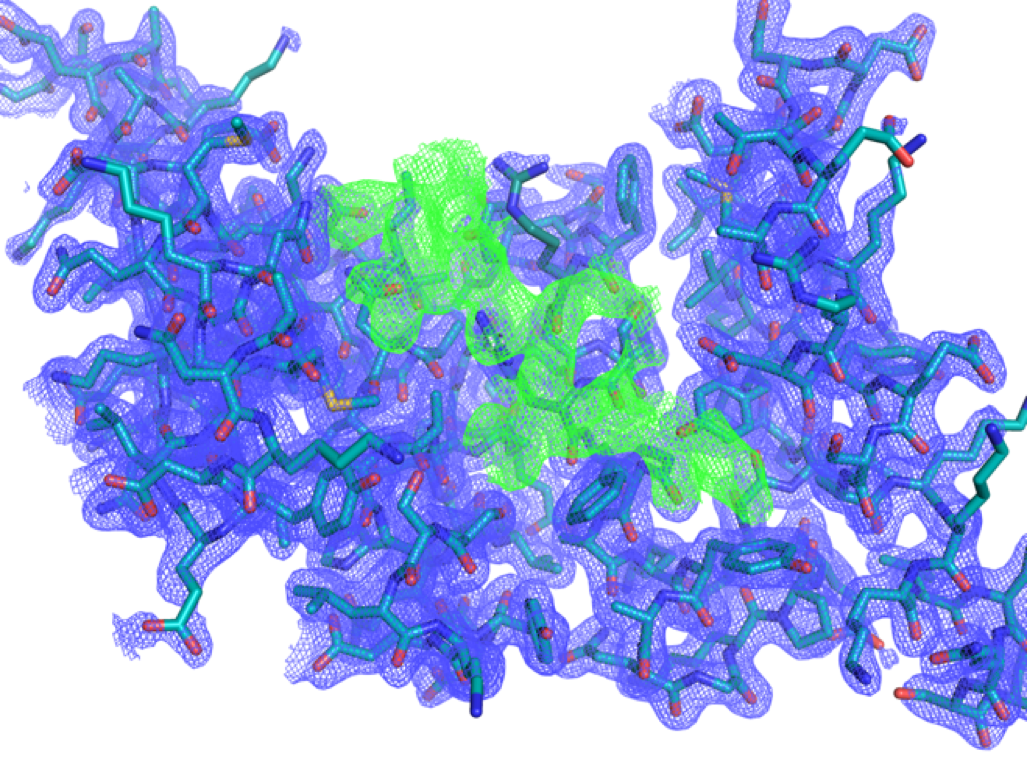


**Figure S2:** Sgt2_TPR grove with 1σ 2Fo-Fc map calculated for the peptide region (green) using the final model from which the peptide was omitted following simulated annealing to remove model bias.


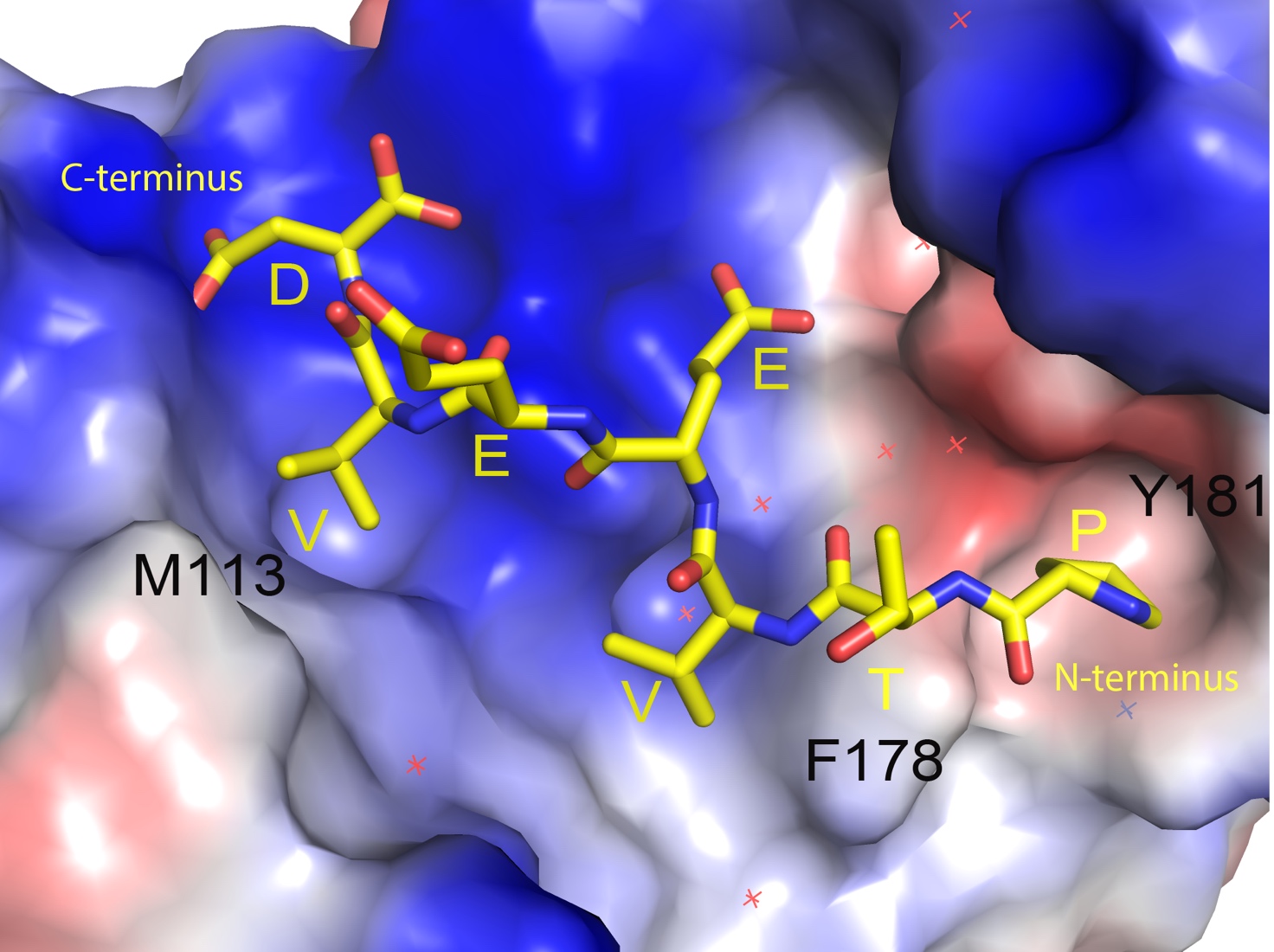


**Figure S3:** Vacuum electrostatics view of Sgt2_TPR hydrophobic pockets highlighting hydrophobic interactions between M113, F178 and Y181 with valine, threonine and proline of the PTVEEVD peptide shown as sticks. The C-terminal end of the peptide is involved in electrostatic interactions. Red corresponds to the negatively charged electrostatic potential on the surface; blue for the positively charged and grey for the neutral. The bound peptides are shown in a stick representation. Red crosses correspond to the water molecules.


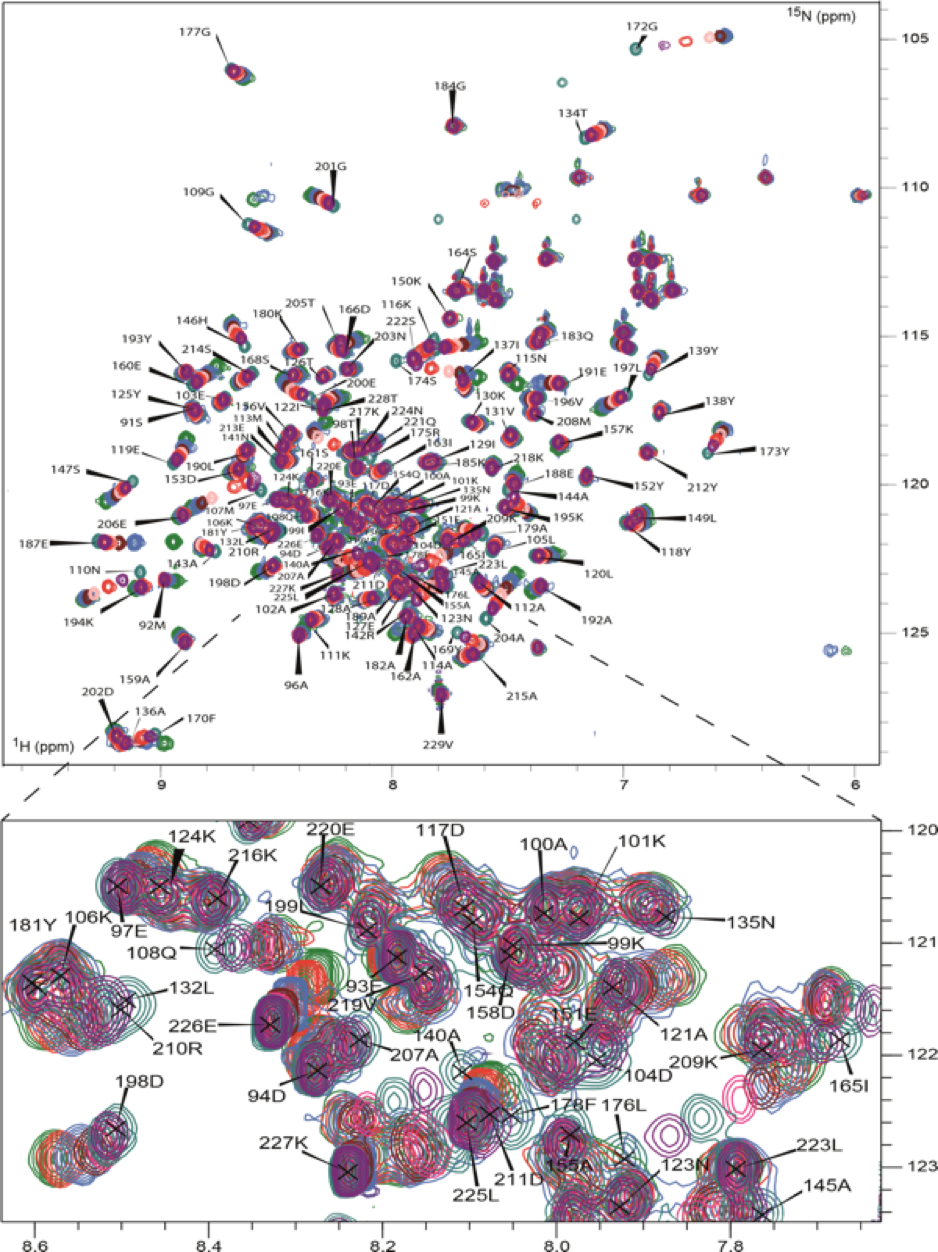


**Figure S4:** The ^1^H-^15^N HSQC spectrum of ^15^N-labelled Sgt2_TPR titrated with unlabelled PTVEEVD at ratios: 1:0 (teal), 1:0.5 (purple), 1:1 (red), 1:2 (light pink), 1:3 (maroon), 1:4 (blue), 1:5 (cyan), and 1:6 (green). The box contains an expansion of the most crowded region of the spectrum. Unassigned peaks encompassing area of ^1^H resonances between 6.0 and 7.5 ppm and ^15^N resonances between 110.0ppm and 115.0 ppm are all side chain amides.


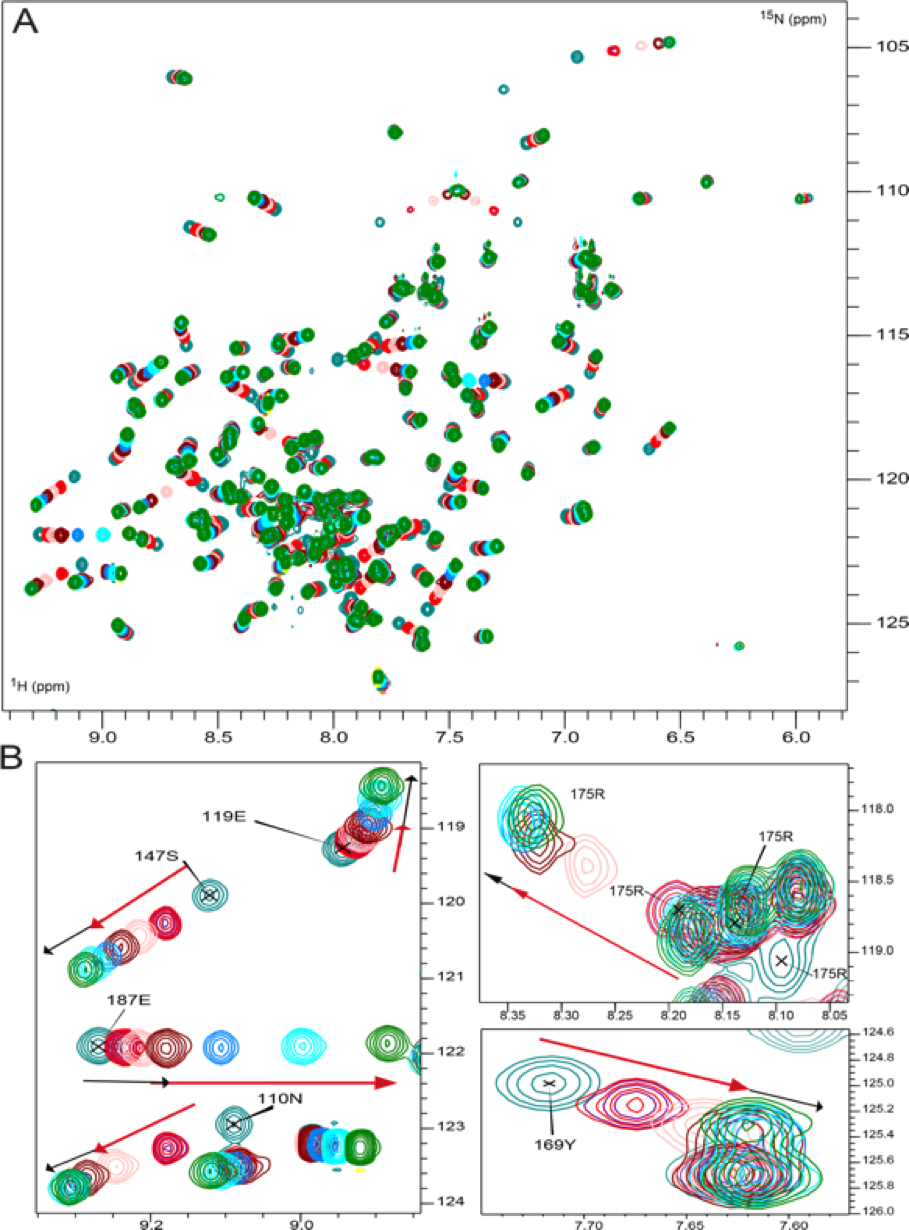


**Figure S5:** A) The ^1^H-^15^N HSQC spectrum of ^15^N-labelled Sgt2_TPR titrated with unlabelled MEEVD at ratios: 1:0 (teal, assigned), 1:0.5 (purple), 1:1 (red), 1:2 (light pink), 1:3 (maroon), 1:4 (blue), 1:5 (cyan), and 1:6 (green). B) Detailed view of ^15^N HSQC spectrum the showing chemical shift perturbation (CSP) for the selected residues. Black and red arrows respectively indicate small and large CSPs upon titration.


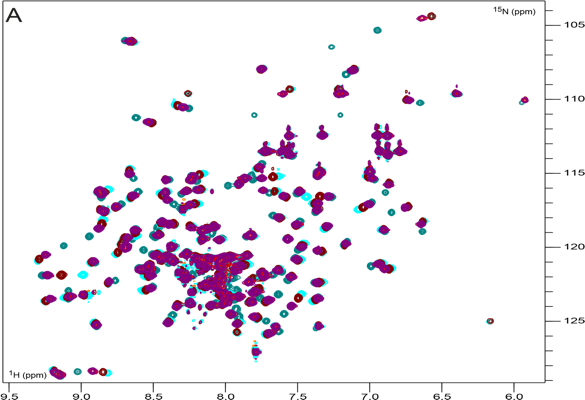


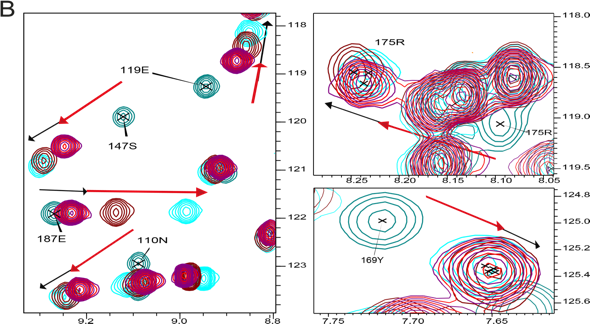


**Figure S6:** A) The ^1^H-^15^N HSQC spectrum of ^15^N-labelled Sgt2_TPR titrated with unlabelled SLEEDLNLD at ratios: 1:0 (teal, assigned), 1:0.5 (purple), 1:1 (red), 1:2 (light pink), 1:3 (maroon), 1:4 (blue), 1:5 (cyan). B) Detailed view of ^15^N HSQC spectrum the showing chemical shift perturbation (CSP) for the selected residues. Black and red arrows indicate small and large CSPs upon titration respectively.


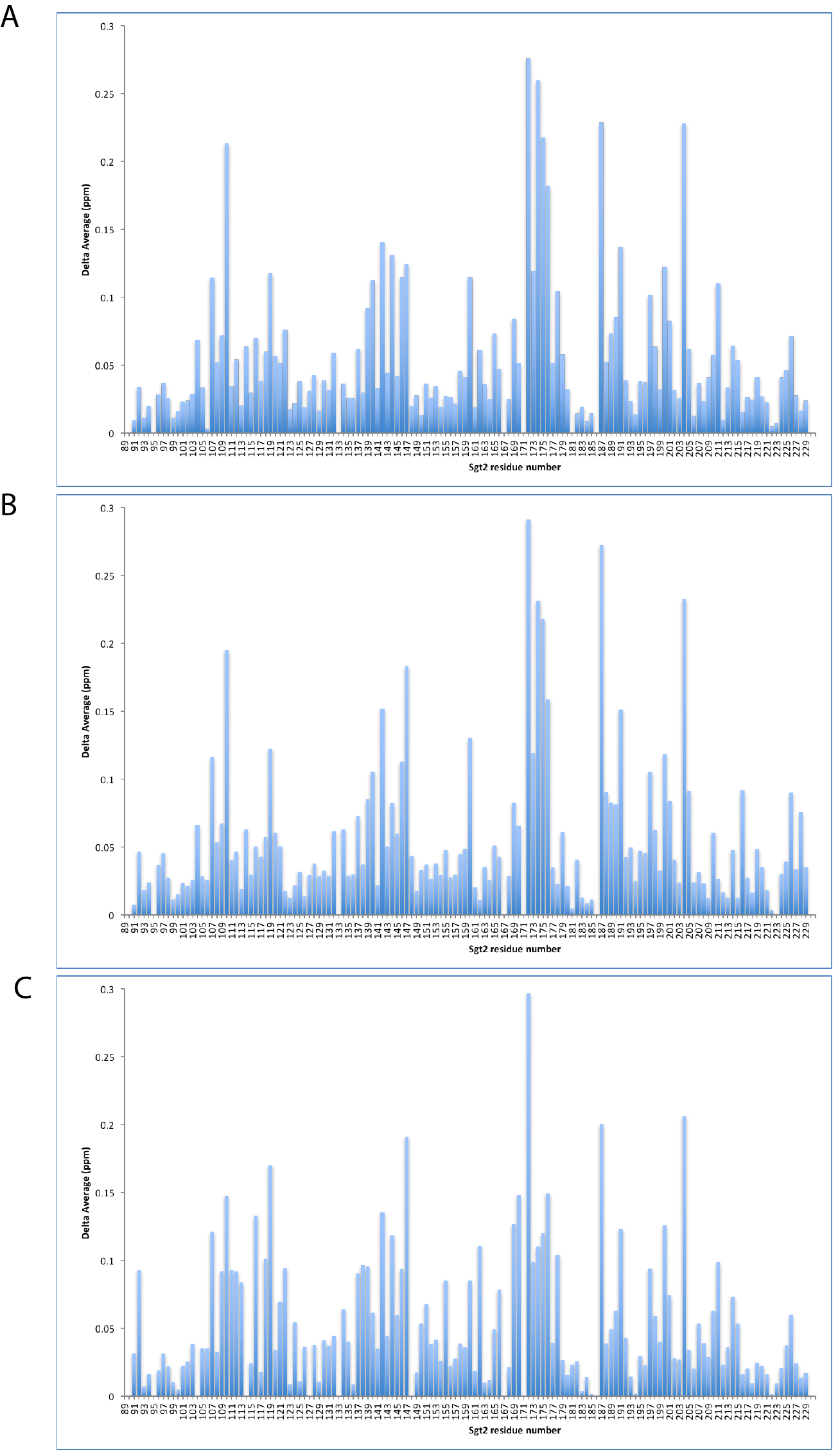


**Figure S7:** Comparison of CSP on a Sgt2 _TPR residue basis between the three NMR titrations with A) PTVEEVD, B) MEEVD and C) SLEEDLNLD.

**Figure S8:** Superimposition of TPR domains from free Sgt2 and Sgt2 bound to Ssa1 derived peptide PTVEEVD (teal). The protein backbones align with RMSD of 0.77 Å over 133 C_α_.
